# Associations of Eating Identities With Self-Reported Dietary Behaviors and Body Mass Index

**DOI:** 10.3389/fnut.2022.894557

**Published:** 2022-07-14

**Authors:** Patrycja Sleboda, Wändi Bruine de Bruin, Lisa Arangua, Tania Gutsche

**Affiliations:** ^1^Schaeffer Center for Health Policy and Economics, University of Southern California, Los Angeles, CA, United States; ^2^Sol Price School of Public Policy, University of Southern California, Los Angeles, CA, United States; ^3^Dornsife Department of Psychology, University of Southern California, Los Angeles, CA, United States; ^4^Center for Economic and Social Research, University of Southern California, Los Angeles, CA, United States; ^5^Los Angeles County Department of Public Health, Los Angeles, CA, United States

**Keywords:** eating identity, dietary behaviors, dietary beliefs, dietary self-efficacy, BMI

## Abstract

**Objective:**

To inform dietary interventions, it is important to understand antecedents of recommended (henceforth: healthy) dietary behaviors, beyond dietary beliefs and self-efficacy. We used the validated “Eating Identity Type Inventory” to assess the extent to which participants identified as healthy eaters, meat eaters, emotional eaters or picky eaters. We examined correlations between participants' race/ethnicity and other socio-demographic characteristics and affinity with these eating identities, how affinity with these eating identities correlated with self-reports of dietary beliefs, self-efficacy, dietary behaviors and Body Mass Index (BMI), and how well affinity with these eating identities predicted self-reported dietary behaviors and BMI, as compared to self-reported dietary beliefs and self-efficacy.

**Methods:**

In an online survey, a diverse sample of 340 Los Angeles County adults reported eating identities, dietary beliefs, and self-efficacy, dietary behaviors and BMI.

**Results:**

Pearson correlations revealed that identifying more as a healthy eater was positively associated with self-reports of being non-Hispanic White, non-Hispanic mixed race, older, and college-educated, while identifying more as a meat eater was positively associated with self-reports of being non-Hispanic Black, younger, and male (α = 0.05). Pearson correlations also showed that healthy eaters had more accurate dietary beliefs and self-efficacy, and emotional eaters had lower self-efficacy (α = 0.05). In linear regressions, identifying more as a healthy eater was associated with self-reporting healthier dietary behaviors and lower BMI, and identifying more as a meat eater and emotional eater was associated with reporting less healthy dietary behaviors and higher BMI, even after accounting for correlations with socio-demographics, dietary beliefs, and self-efficacy (α = 0.05).

**Conclusions:**

Our findings highlight the importance of eating identities in understanding dietary behaviors and outcomes, with implications for dietary interventions.

## Introduction

The Dietary Guidelines for Americans recommends “healthy eating behaviors,” including consuming more fruit and vegetables, and limiting intake of fast food, sweets, sugary beverages and fried food (1, 2). These dietary behaviors are considered healthy for most adults regardless of race/ethnicity, age, or current health status, because of their widespread health benefits (1). Specifically, healthy eating can reduce obesity, which is a risk factor for asthma, diabetes, cardiovascular disease, and some types of cancer (3–5). Healthy dietary behaviors can be individualized to address personal needs, preferences, budgets and cultural traditions (1).

Although the diets of most American adults fail to meet recommendations for healthy eating, there are notable differences between racial/ethnic groups (6–10). Overall, individuals who identify as non-Hispanic Black or Hispanic are least likely to meet the Dietary Guidelines for Americans (7). Individuals who identify as non-Hispanic Black or non-Hispanic White eat less fruit and vegetables than individuals who identify as Hispanic (11), and individuals who identify as Hispanic consume more sugary drinks than non-Hispanic Whites (12). Individuals who identify as non-Hispanic Black consume more processed meat compared to those who identify as non-Hispanic White and Hispanic (13, 14).

According to theories of health behavior change (15), people with more accurate health beliefs about, for example, recommended dietary behaviors, tend to engage more in healthier behaviors (16–20). Meta-analyses have suggested that dietary self-efficacy, or one's perceived ability to stick to a diet (21), is also associated with healthier dietary behaviors, even after accounting for accurate health beliefs (22, 23). Specifically, dietary self-efficacy has been associated with eating more fruit and less fat (24–27). Perhaps as a result, people with greater dietary self-efficacy have better weight control (28, 29).

A growing body of literature has found that it is also important to consider the extent to which people identify with the identity of a healthy eater (30). Self-reported eating identity is a psychosocial concept borrowed from Identity Theory that reflects individuals' perceptions of their own traits and tendencies (31). People who identify themselves more as a healthy eater tend to consume more fruit and vegetables (32, 33), and are more receptive to nutritional interventions that focus on increasing recommended dietary behaviors (30, 32, 34).

Recently, researchers have recognized that four other eating identities, in addition to a healthy-eater identity, are linked to dietary behaviors (35, 36). The validated Eating Identity Type Inventory (37) assesses the degree to which people define themselves as: (a) a *healthy eater* or someone who makes nutritional food choices. (b) a *meat eater* or someone who tends to eat meat. (c) an *emotional eater* or someone who eats more when sad or depressed; and (d) a *picky eater* or someone who avoids specific foods. Each of these four eating identities has been associated with self-reported dietary behaviors (37). Specifically, identifying more as a healthy eater has been associated with self-reports of eating more fruit and vegetables and less fat (37–39). Identifying more as a meat eater has been associated with self-reports of eating less fruit and vegetables and more fat (37), as well as eating more red meat and being less willing to reduce red meat intake (40). Identifying more as an emotional eater or as a picky eater has been associated with self-reports of eating less fruit and vegetables (37).

While research on eating identities shows promise for predicting dietary behaviors, most research on eating identities has focused on the healthy eating identity only (41, 42). Investigations that have focused on the meat, emotional and picky eater identities have been limited, leaving it unclear how these eating identities vary with recommended dietary behaviors (1, 2) other than the intake of fruit and vegetables, fat, and fiber (37, 43). Moreover, while the validated Eating Identity Type Inventory (37) has been used with diverse samples (38, 39), to the best of our knowledge eating identities have not been compared between racial and ethnic groups. To best of our knowledge, no studies have examined the relative importance of these four eating identities for predicting BMI, before and after accounting for dietary beliefs and self-efficacy. To address these research gaps, the present study examined the degree to which a diverse sample of Los Angeles County residents identified as healthy eaters, meat eaters, emotional eaters, and picky eaters to answer the three research questions outlined below:

(1) How do participants' race/ethnicity and other socio-demographic characteristics correlate with their affinity with these eating identities?
(2) How is participants' affinity with these eating identities correlated with dietary beliefs and self-efficacy as well as self-reported dietary behaviors and BMI?
(3) How well does participants' affinity with these eating identities predict self-reported dietary behaviors and BMI, as compared to dietary beliefs and self-efficacy?

## Methods

### Sample and Procedure

Participants aged 18 and older were recruited from Los Angeles County (henceforth LA county) through two sampling strategies. First, participants were recruited through the LA county sample of the Understanding American Study, which was recruited from randomly selected LA county addresses (https://uasdata.usc.edu/page/Recruitment for full details). Second, participants were randomly selected from LA county birth records of the five past years. Invitation letters were addressed to “the family living at.” followed by the specific address. Interested individuals were provided with internet access and tablets as needed. Surveys were offered in English and Spanish, but only 5 of our participants chose to take the Spanish version. Participants provided online consent before starting the online survey. The study protocol was approved by the University of Southern California's Institutional Review Board.

Of the 574 invited participants, 410 (71%) completed the online survey between May 3rd, 2017 and August 31st, 2017. We removed 66 individuals who had missing data on our study variables, and 4 who had biologically implausible values in reported height (<1 meter or <2.5 meters) (37). The final sample included 340 participants aged 19–87 (M = 44.38, SD = 15.43). Demographic characteristics are discussed in the Results section.

### Measures

#### Eating Identity Type Inventory

Participants completed the validated Eating Identity Type Inventory (37). They indicated the extent to which they agreed with 11 statements on a scale from 1 (=completely disagree) to 5 (=completely agree). Three items asked how much they identified as a healthy eater (e.g., I am someone who eats in a nutritious manner). Three items asked how much they identified as a meat eater (e.g., I am a meat eater). Three items asked how much they identified as an emotional eater (e.g., I am someone who eats more when sad/depressed). Two items asked how much they identified as a picky eater (e.g., I am a picky eater). Cronbach's alphas are shown in Table 1.

**Table 1:** Descriptive statistics.

	**Mean (SD) or %**
**Demographics**	
Non-Hispanic white	40.0%
Non-Hispanic black	8.2%
Hispanic	37.9%
Asian	8.8%
Non-Hispanic mixed race	5.0%
Age	M = 44.38 (SD = 15.43)
Male	37.3%
College education	41.2%
Recruited through birth records (vs. addresses)	32.4%
**Self-reported eating identity**	
Healthy eater (1 = strongly disagree; 5 = strongly agree; α = 0.89)	M = 3.51 (SD = 0.9)
Meat eater (1 = strongly disagree; 5 = strongly agree; α = 0.79)	M = 3.13 (SD = 1.23)
Emotional eater (1 = strongly disagree; 5 = strongly agree; α = 0.84)	M = 2.60 (SD = 1.18)
**Self-reported dietary beliefs and self-efficacy**	
Beliefs in healthy diet (1 = not all important; 5 extremely important; α = 0.76)	M = 4.15 (SD = 0.53)
Dietary self-efficacy (1 = I know I cannot; 3 = I know I can; α = 0.94)	M = 2.51 (SD = 0.42)
**Self-reported dietary behaviors**	
Daily fruit and vegetable intake (open-ended)	M = 3.93 (SD = 2.46)
Daily water intake (open-ended)	M = 5.53 (SD = 3.53)
Weekly fast-food frequency (open-ended)	M = 1.33 (SD = 1.25)
Sugary-drinks intake (neve*r =* 0; more than once a day = 3)	M = 1.20 (SD = 1.08)
Never	31.3%
Less frequently than once a day	35.6%
Once a day	14.7%
More than once a day	18.2%
Sweets intake (neve*r =* 0; more than once a day = 3)	M = 1.65 (SD = 0.88)
Never	5.0%
Less frequently than once a day	47.4%
Once a day	25.0%
More than once a day	22.6%
Fried-food intake (neve*r =* 0; more than once a day = 3)	M = 1.09 (SD = 0.60)
Never	8.5%
Less frequently than once a day	80.0%
Once a day	5.9%
More than once a day	5.6%
**Self-reported Body Mass Index**	
Overall BMI	M = 27.70 (SD = 6.22)
Underweight (<18.5)	2.9%
Normal (>18.5 <24.99)	35.6%
Overweight (>24.99 and <30)	32.7%
Obese (>30)	28.8%

#### Dietary Beliefs

Assessments of dietary beliefs were adapted from the Low Income Diet and Nutrition Survey (20). Participants rated the importance of the nine items for a healthy diet (“In your opinion, how important is each of the following for a healthy diet?”), including eating more vegetables and fruits, eating less fat or fatty foods, eating less salt or salty foods, consuming less sugary foods or drinks, eating more fresh or less processed food, eating fewer calories, eating smaller portions, eating more organic food and eating more foods from local farm and local sources. Responses were provided on a scale from 1 (=not at all important) to 5 (=extremely important).

#### Dietary Self-Efficacy

Dietary self-efficacy was assessed through the validated Self-efficacy and Eating Habits Survey (44). Participants rated how hard or easy it is for them to follow twenty healthy dietary behaviors, including eating smaller portions at dinner. The response scale ranged from 1 to 3 (1 = I know I cannot; 2 = Maybe I can; 3 = I know I can). Each item included ‘does not apply' option, which was treated as missing data.

#### Dietary Behaviors

We asked participants about the following dietary behaviors: 1) daily fruit and vegetable intake 2) daily water intake 3) weekly fast-food frequency 4) sugary-drinks intake 5) sweets intake 6) fried-food intake. Dietary guidelines (1, 2) recommend increasing the first two and limiting the others.

*Daily fruit and vegetable intake* was assessed with two open-ended questions adapted from the Special Supplemental Nutrition Program for Women, Infants, and Children in Los Angeles County (45): “on an average day, about how many servings of fruits do you eat? A serving size for most adults is equal to one medium apple” and “on an average day, about how many servings of vegetables do you eat? A serving size for most adults is equal to one handful of broccoli, or a cup of cut carrots.” Fruit and vegetable intake was calculated as the sum of the two responses.

*Daily water intake* was assessed by an open-ended question adapted from the Special Supplemental Nutrition Program for Women, Infants, and Children in Los Angeles County (45): “How often do you drink water? Please give your best guess, choose whatever is easiest in estimating, day, or week” Weekly responses were divided by seven to reflect daily numbers.

*Weekly fast-food frequency* was measured with an open-ended question adapted from the Multi-Ethnic Study of Atherosclerosis (46). Participants were asked “How many times in a typical week do you eat a meal from a fast-food place such as McDonalds's, Taco Bell Subway or similar including take-out and delivery?”

*Sugary drinks intake, sweets intake* and *fried food intake* were assessed by open-ended questions adapted from the Special Supplemental Nutrition Program for Women, Infants, and Children in Los Angeles County (45). Specifically, participants were asked “How often do you drink beverages sweetened with sugar, such as regular sodas Gatorade, Red Bull, and Agua Frescas (Horchata, Jamaica, Tamarindo, Pina). Do not include diet sodas or sugar-free drinks (if necessary:) Please count a 12-ounce can, bottle or glass as one drink.),” “How often do you eat sweets or sweetened foods such as sweetened cereals, fruit bars, pop-tarts, donuts, cookies and candies?” and “How often do you eat deep fried food?” with examples including french fries, fried chicken, fried fish, chicharrones, chicken nuggets, taquitos, and potato chips. Each of these questions was accompanied by the instruction: “Please give your best guess, choose whatever is easiest in estimating, day, week, month, or year. Please enter ‘0' for never.” Response options included 0 = never, 1 = less than once a week, 2 = once a week, 3 = more than once a week.

#### Body Mass Index

Participants were asked to answer open-ended questions about their height (e.g., What is your height?) and their weight (e.g., How much do you approximately weigh?). They could answer in pounds or kilograms for weight and feet and inches or meters and centimeter for height. Responses were transformed to meters and kilograms and BMI was calculated as kilograms divided by squared meters (kg/m2).

### Analyses

All analyses were conducted using SPSS version 25. The one exception was the Confirmatory Factor Analysis on the Eating Identity Type Inventory, which was computed in R (47). For all significance tests, we used two-sided *p*-values (α = 0.05).

Our first preliminary analyses focused on comparing invited individuals who were included in the analyses with those who were not. We conducted a *t*-test to examine group differences in age, and Chi square tests to examine group differences in gender, race/ethnicity, education, and recruitment method through birth records vs. addresses.

Our second preliminary analyses examined the internal consistency of the Eating Identity Type Inventory, including Cronbach's α, inter-item correlations, and Confirmatory Factor Analysis. We retained subscales with Cronbach's alpha ≥.7 and inter-item correlations ≥.3 and acceptable model fit in the Confirmatory Factor Analysis including CFI ≥0.95, TLI ≥0.90, RMSEA ≤ 0.10, and SRMR ≤.08 (48). We also computed Pearson correlations between overall scores reflecting the eating identities for the retained items. Descriptive statistics were computed to reflect the percent of participants identifying with specific race/ethnicity groups, mean (SD) age, the percent of participants identifying as male, college-educated, and recruited through birth records (vs. addresses), as well as the mean (SD) for self-reported eating identity type, dietary self-efficacy and self-reported dietary behaviors and BMI measures (Table 1).

Research question 1 asked about the race/ethnicity and other socio-demographic characteristics of individuals who identified more as a healthy eater, meat eater, emotional eater, or picky eater. To answer Research Question 1, we computed Pearson correlations between participants' reported affinity with these eating identities and their race/ethnicity, age, gender, and education. Additionally, we conducted linear regressions that modeled reported affinity with eating identities as a function of race/ethnicity and other socio- demographic characteristics.

Research question 2 asked how affinity with eating identities was correlated to dietary beliefs, self-efficacy, self-reported dietary behaviors, and BMI. To answer Research Question 2, we computed Pearson's r correlations between eating identities and dietary beliefs, dietary self-efficacy, self-reported dietary behaviors, and BMI.

Research question 3 asked how well participants' affinity with eating identities predicts self-reported dietary behaviors and BMI, as compared to dietary beliefs and self-efficacy. To answer Research Question 3, we compared linear regressions models that predicted self-reported dietary behaviors (daily fruit and vegetable intake, daily water intake, weekly fast-food frequency, sugary-drinks intake, sweets intake, and fried-foods intake) from affinity with eating identities (Model 1 in **Tables 2**–**8**), from dietary beliefs and self-efficacy (Model 2 in **Tables 2**–**8**), and from both (Model 3 in **Tables 2**–**8**). All models treated dependent variables as continuous variables, and took into account race/ethnicity, age, gender, education, and recruitment through birth records vs. addresses. Because sugary-drinks intake, sweets intake and fried food-intake are ordinal variables, we also present ordinal regression models (see Supplemental Material). Assumptions for linearity tend to be robust to multiple methods (49). To facilitate comparisons across dependent variables, we therefore present linear regressions for all dependent variables in the Results section.

## Results

### Preliminary Analyses: Differences Between Included and Excluded Invitees

There were no significant differences between the 340 invitees who were included in the data analyses presented here and the 410 who were not, in terms of the percent identifying as non-Hispanic Black, Asian, non-Hispanic Mixed Race, or their mean age (all *p* > 0.10). However, those who were included in the analyses (vs. not) were significantly more likely to be non-Hispanic White (40.0% vs. 25.2%), χ2 (2) = 13.51, *p* < 0.01, male (37.4% vs. 25.1%), χ2 (2) = 9.42, *p* < 0.01, college-educated (41.2% vs. 27.8%), χ2 (2) = 10.84, *p* < 0.01, as well as less like to be Hispanic (37.9% vs. 59.2%), χ2 (2) = 10.04, *p* < 0.01 and less likely to be recruited through birth records rather than addresses (48.9% vs. 51.1%), χ2 (2) = 16.40, *p* < 0.01.

### Preliminary Analysis: Reliability of Eating Identity Type Inventory

For three of the four eating identities, items had sufficient internal consistency, seen in Cronbach's alpha being at least 0.70. Specifically, Cronbach's alpha was α = 0.89 for identifying as a healthy eater, α = 0.79 for identifying as a meat eater, and α = 0.84 for identifying as an emotional eater. Internal consistency was not sufficient for the picky-eater identity (α = 0.37). As in previous research (37, 40) removing one item with inter-item correlations below 0.30 from the meat-eater subscale (“I am a junk food eater”) improved Cronbach's alpha to 0.79 and inter-item correlations to > 0.65. Based on these findings, we decided to exclude the picky-eater subscale from our analyses, and to remove “I am a junk food eater” from the meat-eater subscale. Doing so did not affect the overall conclusions of our paper.

This decision was supported by Confirmatory Factor Analyses, which was unable to fit all 11 items on four factors representing the healthy eater, meat eater, emotional eater, and picky eater identities. After removing the picky-eater subscale, a Confirmatory Factor Analysis still showed a relatively poor fit of the remaining 8 items on three factors representing the healthy eater, meat eater and emotional eater, seen in CFI ≥0.95, TLI ≥0.90, RMSEA ≤ 0.10 (48), CFI, and SRMR ≤.08 (χ2 (24) = 152.29, *p* < 0.001, CFI = 0.91, TLI = 0.87, RMSEA = 0.12 (90% CI [0.11, 0.15]), SRMR =0.11). After additionally removing “I am a junk food eater” from the meat-eater subscale, we found relatively better model fit (χ2 (17) = 73.72, *p* < 0.001, CFI = 0.96, TLI = 0.93, RMSEA = 0.1 [90% CI (0.08, 0.12)], SRMR = 0.07). Thus, all subsequent analyses were based on the 8 items representing the healthy eater, meat eater, and emotional eater subscales. Mean ratings across included items were computed for each eating identity subscale.

Next, we computed Pearson correlations between mean scores for each eating identity we analyzed. Identifying more as a healthy eater was negatively correlated identifying more as a meat eater (*r* = −0.12, *p* < 0.05) and unrelated to identifying as an emotional eater (*r* = −0.01, *p* = 0.79). Individuals who identified more as a meat eater also identified more as an emotional eater (*r* = 0.20, *p* < 0.05).

### Research Question 1: Race/Ethnicity and Other Socio-Demographic Characteristics of Healthy Eaters, Meat Eaters, Emotional Eaters, and Picky Eaters

Identifying more as a healthy eater was correlated with self-reports of being non-Hispanic White (*r* = 0.11, *p* < 0.05), non-Hispanic Mixed race (*r* = 0.12, *p* < 0.05), and non-Hispanic (*r* = −0.22, *p* < 0.01). Identifying more as a healthy eater was also positively correlated with older age (*r* = 0.18, *p* < 0.01) and having a college degree (*r* = 0.13, *p* < 0.05). Affinity with the healthy-eater identity was not significantly correlated with self-reported non-Hispanic Black or Asian race/ethnicity, or self-reported gender.

Identifying more as a meat eater was correlated with self-reports of being non-Hispanic Black (*r* = 0.13, *p* < 0.05), younger (*r* = −0.23, *p* < 0.01), and male (*r* = 0.11, *p* < 0.05). Affinity with the meat-eater identity was not significantly correlated with self-reports of being non-Hispanic White, Hispanic, Asian, or having a college education.

The degree to which participants identified as an emotional eater was positively correlated with self-reports of being non-Hispanic mixed race (*r* = 0.11, *p* < 0.05) and negatively correlated with self-reports of being non-Hispanic White (*r* = −0.11, *p* < 0.05). Affinity with the emotional-eater identity was not significantly correlated with other race/ethnicity groups (non-Hispanic Black, Hispanic, or Asian), age, gender, or education.

### Research Question 2: Correlations of Eating Identities With Self-Reported Dietary Behaviors, BMI, Dietary Beliefs, and Self-Efficacy

Identifying more as a healthy eater was correlated with self-reports of eating more fruit and vegetables (*r* = 0.30, *p* < 0.01), drinking more water (*r* = 0.16, *p* < 0.01), less frequent dining in fast-food restaurants (*r* = −0.35, *p* < 0.01), and lower intake of sugary drinks (r = −0.29, *p* < 0.01), sweets (r = −0.24, *p* < 0.01), and fried food (*r* = −0.22, *p* < 0.01). Participants who identified more as a healthier eater also reported lower BMI (*r* = −0.34, *p* < 0.01), had more accurate beliefs about healthy diets (*r* = 0.12, *p* < 0.05), and had greater dietary self-efficacy (r=0.18, *p* < 0.01).

Identifying more as a meat eater was associated with self-reporting more frequent fast-food dining (*r* = 0.22, *p* < 0.01), consuming more sugary drinks (*r* = 0.19, *p* < 0.01), eating more fried food (*r* = 0.16, *p* < 0.01) and higher BMI (*r* = 0.12, *p* < 0.05). Identifying more as a meat eater was associated with lower dietary self-efficacy (*r* = –0.27, *p* < 0.01). Affinity with the meat-eater identity showed no significant relationship with dietary beliefs, self-reported intake of fruit and vegetables, water, or sweets.

Identifying more as an emotional eater was related to more self-reported consumption of sugary drinks (*r* = 0.15, *p* < 0.01), sweets (*r* = 0.13, *p* < 0.05), and fried food (*r* = 0.17, *p* < 0.01). Participants who identified more as emotional eaters also reported higher BMI (*r* = 0.24, *p* < 0.01), and lower dietary self-efficacy (*r* = –0.23, *p* < 0.01). Affinity with the emotional-eater identity was unrelated to dietary beliefs, or the consumption of fruit and vegetables, water, fast food.

### Research Question 3: Eating Identities' Ability to Predict Self-Reported Dietary Behaviors and BMI, as Compared to Dietary Beliefs and Self-Efficacy

Most of the relationships of eating identities with self-reported dietary behaviors and BMI that were reported for Research Question 2 still held in linear regressions that controlled for dietary self-efficacy, dietary beliefs, and socio-demographics (**Tables 2**–**8**, Model 3). Identifying more as a healthy eater was the best predictor of self-reporting more fruit and vegetables intake (β = 0.32; *p* < 0.001; Table 2, Model 3) and was also associated with more self-reported daily water drinking (β = 0.19; *p* < 0.001; Table 3, Model 3). Identifying less as a healthy eater (β = −28; *p* < 0.001) and more as a meat eater (β = 0.21; *p* < 0.001) was associated with more self-reported fast-food dining (Table 4, Model 3).

**Table 2:** Linear regressions predicting self-reported daily fruit and vegetable intake.

	**Dependent variable: self–reported daily fruit and vegetable intake^a^**					
	**Model 1**		**Model 2**		**Model 3**	
**Predictor variables**	Adj. R2 = 0.11 F(11,339) = 4.67^***^		Adj. R2 = 0.01 F(10,339) = 1.52 p = 0.13		Adj. R2 = 0.11 F(13,339) = 4.12^***^	
	**β**	**[95% CI]**	**β**	**[95% Cl]**	**β**	**[95% Cl]**
Healthy eater	0.31^***^	**(0.55, 1.13)**			0.32^***^	**(0.57, 1.17)**
Meat eater	−0.07	(−0.36, 0.08)			−0.07	(−0.37, 0.09)
Emotional eater	0.04	(−0.14, 0.29)			0.03	(−0.15, 0.29)
Dietary beliefs^b^			−0.03	(−0.65, 0.35)	−0.08	(−0.84, 0.13)
Dietary self–efficacy^c^			0.07	(−0.23, 1.05)	0.00	(−0.62, 0.67)
						(−0.84, 0.13)
Demographics						
Non–Hispanic white	0.00	(−1.21, 1.24)	−0.11	(−1.81, 0.73)	0.00	(−1.22, 1.24)
Non–Hispanic black	0.04	(−1.11, 1.79)	−0.03	(−1.77, 1.28)	0.03	(−1.15, 1.77)
Hispanic	−0.02	(−1.41, 1.17)	−0.17	(−2.18, 0.48)	−0.02	(−1.40, 1.19)
Asian	0.00	(−1.38, 1.41)	−0.04	(−1.81, 1.13)	0.01	(−1.35, 1.46)
Age	−0.05	(−0.03, 0.01)	0.01	(−0.02, 0.02)	−0.05	(−0.03, 0.01)
Male	−0.15^*^	**(−1.32**, **−0.18)**	−0.13^*^	**(−1.27**, **−0.09)**	−0.15^*^	**(−1.32**, **−0.18)**
College education	0.04	(−0.37, 0.81)	0.04	(−0.40, 0.84)	0.03	(−0.43, 0.76)
Recruited through birth						
records (vs. addresses)	0.07	(−0.30, 1.07)	0.12	(−0.09, 1.34)	0.08	(−0.28, 1.09)

*Statistically significant in bold font.*

* *p < 0.05;*

^**^p < 0.01;

*** *p < 0.001.*

a *answered in an open ended question.*

b *average response across 9 items on a 1–5 scale.*

c *average response across 20 items on a 1–3 scale*.

**Table 3:** Linear regressions predicting self–reported daily water intake.

	**Dependent variable: self–reported daily water intake^a^**					
	**Model 1**		**Model 2**		**Model 3**	
**Predictor variables**	Adj. R2 = 0.10 F(11,339) = 4.25^***^		Adj. R2 = 0.07 F(10,339) = 3.39^***^		Adj. R2 = 0.10 F(13,339) = 3.81^***^	
	**β**	**[ 95% Cl]**	**β**	**[ 95% Cl]**	**β**	**[ 95% Cl]**
Healthy eater	0.20^***^	**(0.35, 1.18)**			0.19^***^	**(0.31, 1.17)**
Meat eater	0.11	(−0.01, 0.63)			0.10	(−0.03, 0.62)
Emotional eater	0.03	(−0.22, 0.41)			0.03	(−0.24, 0.40)
Dietary beliefs^b^			0.10	(−0.02, 1.38)	0.08	(−0.15, 1.24)
Dietary self–efficacy^c^			−0.03	(−1.17, 0.62)	−0.03	(−1.23, 0.64)
Demographics						
Non–Hispanic white	−0.15	(−2.87, 0.67)	−0.21	(−3.28, 0.28)	−0.14	(−2.82, 0.73)
Non–Hispanic black	−0.13	(−3.74, 0.45)	−0.14	(−3.90, 0.35)	−0.12	(−3.63, 0.59)
Hispanic	−0.17	(−3.12, 0.60)	−0.27^*^	**(−3.83**, **−0.12)**	−0.17	(−3.09, 0.65)
Asian	−0.06	(−2.77, 1.27)	−0.08	(−3.09, 1.03)	−0.06	(−2.78, 1.28)
Age	−0.13	(−0.06, 0.00)	−0.13^*^	**(−0.06, 0.00)**	−0.13	(−0.06, 0.00)
Male	−0.23^***^	**(−2.50**, **−0.86)**	−0.20^***^	**(−2.30**, **−0.66)**	−0.23^***^	**(−2.51**, **−0.87)**
College education	0.09	(−0.24, 1.46)	0.12	(−0.04, 1.70)	0.10	(−0.15, 1.56)
Recruited through birth records (vs. addresses)	−0.04	(−1.27, 0.70)	−0.02	(−1.13, 0.86)	−0.04	(−1.32, 0.65)

*Statistically significant in bold font.*

* *p < 0.05;*

^**^p < 0.01;

*** *p < 0.001.*

a *answered in an open ended question.*

b *average response across 9 items on a 1–5 scale.*

c *average response across 20 items on a 1–3 scale*.

**Table 4:** Linear regressions predicting self–reported weekly fast–food frequency.

	**Dependent variable: self–reported weekly fast–food frequency^a^**					
	**Model 1**		**Model 2**		**Model 3**	
**Predictor variables**	Adj. R2 = 0.20 F(11,339) = 8.72^***^		Adj. R2 = 0.09 F(10,339) = 4.31^***^		Adj. R2 = 0.20 F(13,339) = 7.56^***^	
	**β**	**[95% Cl]**	**β**	**[95% Cl]**	**β**	**[95% Cl]**
Healthy eater	−0.27^***^	**(−0.51**, **−0.23)**			−0.28^***^	**(−0.53, 0.24)**
Meat eater	0.19^***^	**(0.09, 0.30)**			0.21^***^	**(0.10, 0.32)**
Emotional eater	0.04	(−0.06, 0.15)			0.05	(−0.05, 0.16)
Dietary beliefs^b^			−0.05	(−0.36, 0.13)	0.00	(−0.24, 0.22)
Dietary self–efficacy^c^			−0.03	(−0.40, 0.22)	0.08	(−0.07, 0.55)
Demographics						
Non–Hispanic white	−0.01	(−0.60, 0.57)	0.06	(−0.48, 0.76)	−0.02	(−0.65, 0.53)
Non–Hispanic black	0.04	(−0.51, 0.88)	0.09	(−0.32, 1.17)	0.03	(−0.58, 0.82)
Hispanic	0.19	(−0.14, 1.10)	0.26^***^	**(0.02, 1.32)**	0.16	(−0.20, 1.05)
Asian	−0.01	(−0.73, 0.61)	0.01	(−0.66, 0.78)	−0.03	(−0.80, 0.55)
Age	−0.07	(−0.02, 0.00)	−0.18^*^	**(−0.02, 0.00)**	−0.07	(−0.02, 0.00)
Male	0.04	(−0.16, 0.39)	0.06	(−0.14, 0.43)	0.05	(−0.15, 0.40)
College education	−0.14^*^	**(−0.63**, **−0.07)**	−0.14^*^	**(−0.66**, **−0.05)**	−0.14^*^	**(−0.65**, **−0.08)**
Recruited through birth records (vs. addresses)	−0.09	(−0.58, 0.08)	−0.12	(−0.67, 0.03)	−0.09	(−0.57, 0.09)

*Statistically significant in bold font.*

* *p < 0.05;*

^**^p < 0.01;

*** *p < 0.001.*

a *answered in an open ended question.*

b *average response across 9 items on a 1–5 scale.*

c *average response across 20 items on a 1–3 scale*.

When controlling for race, age, gender, and education, identifying less as a healthy eater (β = −0.18; *p* < 0.001), more as a meat eater (β = 0.12; *p* < 0.05), and more as an emotional eater (β = 0.10; *p* < 0.05) was significantly related to self-reported sugary drinks consumption (Table 5, Model 1). Similar results were obtained in ordinal regressions (Supplementary Table A2, Model 1). When controlling for dietary self-efficacy and dietary beliefs the predictive power of meat and emotional eating identities were no longer significant in the linear regression (Table 5 Model 3) though they were significant in the ordinal regression (Supplementary Table A2, Model 3).

**Table 5:** Linear regressions predicting self–reported sugary–drinks intake.

	**Dependent variable: self–reported sugary–drinks intake^a^**					
	**Model 1**		**Model 2**		**Model 3**	
**Predictor variables**	Adj. R2 = 0.26 F(11,339) = 11.86^***^		Adj. R2 = 0.22 F(10,339) = 10.71^***^		Adj. R2 = 0.26 F(13,339) = 10.26^***^	
	**β**	**[ 95% Cl]**	**β**	**[95% Cl]**	**β**	**[95% Cl]**
Healthy eater	−0.18^***^	**(−0.33**, **−0.10)**			−0.17^***^	**(−0.32**, **−0.08)**
Meat eater	0.12^*^	**(0.02, 0.20)**			0.11	(0.00, 0.18)
Emotional eater	0.10^*^	**(0.01, 0.18)**			0.09	(−0.01, 0.17)
Dietary beliefs^a^			−0.05	(−0.30, 0.09)	−0.02	(−0.24, 0.14)
Dietary self–efficacy^b^			−0.15^***^	**(−0.63**, **−0.14)**	−0.08	(−0.45, 0.06)
Demographics						
Non–Hispanic white	−0.05	(−0.60, 0.37)	0.00	(−0.49, 0.49)	−0.03	(−0.56, 0.41)
Non–Hispanic black	−0.01	(−0.63, 0.52)	0.03	(−0.46, 0.72)	0.00	(−0.59, 0.57)
Hispanic	0.07	(−0.36, 0.66)	0.14	(−0.21, 0.82)	0.09	(−0.31, 0.72)
Asian	0.00	(−0.57, 0.54)	0.03	(−0.45, 0.70)	0.01	(−0.52, 0.60)
Age	−0.21^***^	**(−0.02**, **−0.01)**	−0.26^***^	**(−0.03**, **−0.01)**	−0.21^***^	**(−0.02**, **−0.01)**
Male	0.01	(−0.21, 0.24)	0.00	(−0.23, 0.23)	0.00	(−0.22, 0.23)
College education	−0.18^***^	**(−0.63**, **−0.16)**	−0.18^***^	**(−0.63**, **−0.15)**	−0.18^***^	**(−0.63**, **−0.16)**
Recruited through birth records (vs. addresses)	0.07	(−0.11, 0.43)	0.05	(−0.15, 0.40)	0.07	(−0.12, 0.43)

*Statistically significant in bold font.*

* *p < 0.05;*

^**^p < 0.01;

*** *p < 0.001.*

a *answered in an open ended question.*

b *average response across 9 items on a 1–5 scale.*

*^c^average response across 20 items on a 1–3 scale*.

Identifying less as a healthy eater (β = −0.21; *p* < 0.001) and more as an emotional eater (β = 0.12; *p* < 0.05) was associated with self-reporting more sweets consumption, which was seen in linear regression (Table 6, Model 3) and ordinal regressions (Supplementary Table A3, Model 3).

**Table 6:** Linear regressions predicting self–reported sweets intake.

	**Dependent variable: self–reported sweets intake^a^**					
	**Model 1**		**Model 2**		**Model 3**	
**Predictor variables**	Adj. R2 = 0.08 F(11,339) = 3.69^***^		Adj. R2 = 0.03 F(10,339) = 2.21^*^		Adj. R2 = 0.08 F(13,339) = 3.21^***^	
	**β**	**[ 95% Cl]**	**β**	**[ 95% Cl]**	**β**	**[ 95% Cl]**
Healthy eater	−0.22^***^	**(−0.32**, **−0.11)**			−0.21^***^	**(−0.31**, **−0.10)**
Meat eater	0.01	(−0.07, 0.09)			0.00	(−0.08, 0.08)
Emotional eater	0.13^*^	**(0.02, 0.18)**			0.12^*^	**(0.01, 0.17)**
Dietary beliefs^b^			−0.06	(−0.29, 0.07)	−0.03	(−0.23; 0.12)
Dietary self–efficacy^c^			−0.12^*^	**(−0.47**, **−0.02)**	−0.05	(−0.35, 0.12)
Demographics						
Non–Hispanic white	0.26^*^	**(0.02, 0.91)**	0.31^*^	**(0.10, 1.01)**	0.27^*^	**(0.04, 0.94)**
Non–Hispanic black	0.06	(−0.35, 0.71)	0.09	(−0.24, 0.84)	0.07	(−0.32, 0.74)
Hispanic	0.13	(−0.24, 0.70)	0.21	(−0.08, 0.86)	0.15	(−0.21, 0.74)
Asian	0.09	(−0.23, 0.79)	0.12	(−0.14, 0.91)	0.10	(−0.20, 0.83)
Age	−0.05	(−0.01, 0.00)	−0.08	(−0.01, 0.00)	−0.05	(−0.01, 0.00)
Male	0.01	(−0.19, 0.22)	−0.01	(−0.23, 0.19)	0.01	(−0.20, 0.22)
College education	−0.01	(−0.24, 0.19)	−0.02	(−0.26, 0.18)	−0.02	(−0.24, 0.19)
Recruited through birth records (vs. addresses)	0.15^*^	**(0.02, 0.52)**	0.13	(−0.01, 0.49)	0.14^*^	**(0.02, 0.52)**

*Statistically significant in bold font.*

* *p < 0.05;*

^**^p < 0.01;

*** *p < 0.001.*

a *answered in an open ended question.*

b *average response across 9 items on a 1–5 scale.*

c *average response across 20 items on a 1–3 scale*.

In linear regressions predicting self-reported fried food consumption, identifying less as a healthy eater (β = −0.14; *p* < 0.05) and more as an emotional eater (β = 0.12; *p* < 0.05) predicted more fried-food intake but meat-eating identity was not significantly associated (Table 7, Model 3). Ordinal regression models predicting self-reported fried food consumption revealed the significant relationship for healthy-eater identity but not for meat-eater nor emotional-eater identities (Supplementary Table A4, Model 3).

**Table 7:** Linear regressions predicting self–reported fried–food intake.

	**Dependent variable: self–reported fried food intake^a^**					
	**Model 1**		**Model 2**		**Model 3**	
**Predictor variables**	Adj. R2 = 0.11 F(11,339) = 4.92^***^		Adj. R2 = 0.09 F(10,339) = 4.27^***^		Adj. R2 = 0.12 F(13,339) = 4.42^***^	
	**β**	**[95% Cl]**	**β**	**[95% Cl]**	**β**	**[95% Cl]**
Healthy eater	−0.16^***^	**(−0.17**, **−0.03)**			−0.14^*^	**(−0.16**, **−0.02)**
Meat eater	0.07	(−0.02, 0.09)			0.05	(−0.03, 0.08)
Emotional eater	0.14^*^	**(0.02, 0.13)**			0.12^*^	**(0.01, 0.12)**
Dietary beliefs^b^			−0.04	(−0.16, 0.07)	−0.01	(−0.13, 0.10)
Dietary self–efficacy^c^			−0.16^***^	**(−0.38**, **−0.08)**	−0.10	(−0.29, 0.02)
Demographics						
Non–Hispanic white	0.00	(−0.30, 0.29)	0.03	(−0.26, 0.34)	0.02	(−0.28, 0.32)
Non–Hispanic black	0.02	(−0.30, 0.40)	0.06	(−0.23, 0.49)	0.04	(−0.27, 0.44)
Hispanic	0.04	(−0.26, 0.37)	0.10	(−0.19, 0.44)	0.07	(−0.23, 0.40)
Asian	−0.02	(−0.38, 0.30)	0.01	(−0.32, 0.37)	0.00	(−0.35,0.34)
Age	−0.17^*^	**(−0.01, 0.00)**	−0.21^***^	**(−0.01, 0.00)**	−0.17^*^	**(−0.01, 0.00)**
Male	0.01	(−0.13, 0.15)	0.00	(−0.14, 0.13)	0.00	(−0.13, 0.14)
College education	−0.04	(−0.20, 0.09)	−0.04	(−0.20, 0.09)	−0.04	(−0.19, 0.09)
Recruited through birth records (vs. addresses)	0.04	(−0.11, 0.22)	0.03	(−0.13, 0.21)	0.04	(−0.12, 0.22)

*Statistically significant in bold font.*

* p < 0.05; **p < 0.01;

*** *p < 0.001.*

a *answered in an open ended question.*

b *average response across 9 items on a 1–5 scale.*

c *average response across 20 items on a 1–3 scale*.

Finally, higher BMI (Table 8, Model 3) was associated with identifying less as a healthy eater (β = −0.31; *p* < 0.001), more as a meat eater (β = 0.13; *p* < 0.05) and more as an emotional eater (β = 0.25; *p* < 0.001).

**Table 8:** Linear regressions predicting Body Mass Index.

	**Dependent variable: self–reported Body Mass Index^a^**					
	**Model 1**		**Model 2**		**Model 3**	
Predictor variables	Adj. R2 = 0.29 F(11,339) = 13.47^***^		Adj. R2 = 0.14 F(10,339) = 6.61^***^		Adj. R2 = 0.31 F(13,339) = 12.63^***^	
	β	[95% Cl]	β	[95% Cl]	β	[95% Cl]
Healthy eater	−0.28^***^	**(−2.58**, **−1.28)**			−0.31^***^	**(−2.79**, **−1.47)**
Meat eater	0.11^*^	**(0.08, 1.08)**			0.13^*^	**(0.14, 1.15)**
Emotional eater	0.24^***^	**(0.78, 1.76)**			0.25^***^	**(0.85, 1.84)**
Dietary beliefs^b^			0.10	(−0.03, 2.33)	0.15^***^	**(0.71, 2.86)**
Dietary self–efficacy^c^			−0.10	(−3.00, 0.01)	0.04	(−0.85, 2.02)
Demographics						
Non–Hispanic white	0.00	(−2.70, 2.82)	0.03	(−2.60, 3.40)	−0.01	(−2.80, 2.66)
Non–Hispanic black	0.09	(−1.13, 5.41)	0.15	(−0.31, 6.86)	0.09	(−1.13, 5.37)
Hispanic	0.28^*^	**(0.64, 6.44)**	0.33^*^	**(1.11, 7.37)**	0.26^*^	**(0.41, 6.17)**
Asian	−0.12	(−5.75, 0.54)	−0.10	(−5.67, 1.26)	−0.14	(−6.12, 0.14)
Age	0.21^***^	**(0.04, 0.13)**	0.13^*^	**(0.00, 0.10)**	0.21^***^	**(0.04, 0.13)**
Male	0.07	(−0.33, 2.23)	0.07	(−0.55, 2.23)	0.08	(−0.27, 2.26)
College education	−0.13^*^	**(−3.02**, **−0.37)**	−0.12^*^	**(−2.96**, **−0.03)**	−0.12^*^	**(−2.78**, **−0.14)**
Recruited through birth records (vs. addresses)	0.00	(−1.59, 1.49)	−0.03	(−2.07, 1.30)	−0.01	(−1.66, 1.38)

*Statistically significant in bold font.*

* *p < 0.05;*

^**^p < 0.01;

*** *p < 0.001.*

a *answered in an open ended question.*

b *average response across 9 items on a 1–5 scale.*

c *average response across 20 items on a 1–3 scale*.

Overall, these regressions suggest that eating identities were the stronger predictors of self-reported dietary behaviors, as compared to dietary beliefs and self-efficacy. Neither models with dietary beliefs and self-efficacy alone nor full models that included eating identities together with dietary beliefs and self-efficacy had larger predictive power (Adjusted R^2^) than models with eating identities alone (**Tables 2**–**8**, Models 1 and 3 vs. Model 2).

## Discussion

The Dietary Guidelines for Americans suggest that for most people, eating healthy can help in fighting obesity and chronic disease risk (1). Interventions that aim to promote healthy dietary behaviors need to build on reliable predictors of these healthy dietary behaviors. Here, we therefore examined eating identities in a diverse LA county sample and reported on three main findings.

First, due to our diverse Los Angeles County country sample, we were able to report how eating identities varied by race/ethnicity and other socio-demographic differences. Specifically, identifying as a healthy eater was seen more among individuals who self-reported being non-Hispanic White and non-Hispanic Mixed Race, and less among Hispanic participants. Moreover, identifying more as a meat eater was more common among non-Hispanic Black participants. We also found that identifying more as a healthy eater was associated with being older and college-educated, while identifying as a meat eater was associated with being younger and male. These results can potentially help to tailor interventions to specific socio-demographic needs. As noted by the Dietary Guidelines for Americans, healthy dietary behaviors can be individualized to address personal preferences and cultural traditions (1).

Our second finding was that eating identities were associated with dietary beliefs, dietary self-efficacy, and self-reported dietary behaviors. Previous research had already shown that identifying more as a healthy eater was positively associated with accurate dietary beliefs (41), greater dietary self-efficacy (32) and more fruit and vegetable intake (32, 37). Here, we found that identifying more as a healthy eater was also associated with reporting other healthy dietary behaviors, including drinking more water, avoiding fast foods, sugary drinks, sweets, and fried foods, as well as reporting lower BMI. Moreover, we found that identifying more as a meat eater or emotional eater was associated with reporting less dietary self-efficacy, eating more unhealthy food, and higher BMI–while being uncorrelated with dietary beliefs.

Our third main finding was that eating identities were better predictors of self-reported dietary behaviors and BMI as compared to dietary self-efficacy and dietary beliefs, while taking into account socio-demographic characteristics. Our findings have a potential implication for interventions that aim to promote healthy diets. Indeed, interventions may be more effective if they address people's eating identities, including those related to healthy eating, meat eating, and emotional eating.

The study has four main limitations. First, all dependent variables were self-reported, which may undermine their validity. Self-reported dietary behaviors may be more valid when using diary methods (50, 51). Self-reported BMIs tend to be underestimates of actual BMI (52). However, self-reported BMIs do correlate highly with technician-measured BMI, independent of age, sex, ethnicity, or obesity status (53). Second, the meat-eater subscale of the Eating Identity Type Inventory does not distinguish between eating red and lean meat, even though the latter is usually healthier than the former. Third, the presented study is cross-sectional. To examine casual effects of eating identities on outcome variables, we would need to randomly assign participants to interventions that promote specific eating identities, and then examines effects on dietary behaviors and BMI. Furthermore, our sample included only residents of Los Angeles County, who may be diverse but are not necessarily representative of the United States population.

Despite these limitations, our study has implications for dietary interventions. Specifically, health interventions that aim to improve dietary behaviors could benefit from focusing on encouraging people to identify more as a healthy eater, less as a meat eater, and less as an emotional eater. Text-message reminders have been found to increase identifying as a healthy eater while decreasing identifying as a meat eater–which helped to promote healthier dietary behaviors (40). Eating identities may also be promoted by highlighting descriptive social norms, which is more effective than dietary information (54). Robinson and collaborators (54) found that students intake of fruit and vegetables significantly increased when presented with message about peers dietary behaviors as compared to health messages. Similar interventions could focus both social norms and healthy-eater identity. Eating identities may also play a role in responsiveness to interventions such as calorie labeling (55). Future studies could focus on exploring these findings in other samples in experimental settings including experience sampling methodology to track associations with eating identity on a daily level.

## Data Availability Statement

The data underlying this article are publicly available: https://uasdata.usc.edu.

## Ethics Statement

This study involved human participants. It was reviewed and approved by University of Southern California's Institutional Review Board. The participants provided their written informed consent to participate in this study.

## Author Contributions

PS, WB, LA, and TG: conceptualization and methodology. PS: formal analysis and writing—original draft. WB: supervision. WB and PS: writing—review and editing. LA, WB, and TG: funding acquisition. All authors have approved the final manuscript.

## Funding

Data collection was funded by the LA Department of Public Health and First Five LA as part of their Choose Health LA program, as well as the Dornsife Center for Economic and Social Research. Wändi Bruine de Bruin was partially funded by the University of Southern California's Schaeffer Center for Health Policy and Economics.

## Conflict of Interest

The authors declare that the research was conducted in the absence of any commercial or financial relationships that could be construed as a potential conflict of interest.

## Publisher's Note

All claims expressed in this article are solely those of the authors and do not necessarily represent those of their affiliated organizations, or those of the publisher, the editors and the reviewers. Any product that may be evaluated in this article, or claim that may be made by its manufacturer, is not guaranteed or endorsed by the publisher.

## References

[1] U.S. Department of Agriculture and U.S. Department of Health and Human Services. Dietary Guidelines for Americans, 2020-2025. (2020). Available online at: DietaryGuidelines.gov (accessed June, 2022).

[2] Center for Disease Control Prevention. Healthy Eating for a Healthy Weight. (2021). Available online at: https://www.cdc.gov/healthyweight/healthy_eating/ (accessed June, 2022).

[3] Afshin A Sur PJ Fay KA Cornaby L Ferrara G Salama JS. Health effects of dietary risks in 195 countries, 1990–2017: a systematic analysis for the global burden of disease study 2017. Lancet. (2019) 393:1958–72. 10.1016/S0140-6736(19)30041-8 30954305 PMC6899507

[4] Michelson PH Williams LW Benjamin DK Barnato AE. Obesity, inflammation, and asthma severity in childhood: data from the National Health and Nutrition. Ann Allergy, Asthma Immunol. (2009) 103:381–5. 10.1016/S1081-1206(10)60356-0 19927535

[5] Hales CM Carroll MD Fryar CD Ogden CL Ph D. Prevalence of obesity and severe obesity among adults: United States, 2017–2018. NCHS Data Brief. (2020) (360):1–7. 32487284

[6] August KJ Sorkin DH. Racial/ethnic disparities in exercise and dietary behaviors of middle-aged and older adults. J Gen Intern Med. (2011) 26:245–50. 10.1007/s11606-010-1514-7 20865342 PMC3043172

[7] Kirkpatrick SI Dodd KW Reedy J Krebs-Smith SM. Income and race/ethnicity are associated with adherance to food-based dietary guidance among U. S adults and children J Acad Nutr Diet. (2012) 112:624–35. 10.1016/j.jand.2011.11.012 22709767 PMC3775640

[8] Baraldi LG Martinez Steele E Canella DS Monteiro CA. Consumption of ultra-processed foods and associated sociodemographic factors in the USA between 2007 and 2012: evidence from a nationally representative cross-sectional study. BMJ Open. (2018) 8:20574 10.1136/bmjopen-2017-020574 29525772 PMC5855172

[9] Sorkin DH Billimek J. Dietary behaviors of a racially and ethnically diverse sample of overweight and obese californians. Heal Educ Behav. (2012) 39:737–44. 10.1177/1090198111430709 22467636 PMC5769478

[10] Moore L V Dodd KW Thompson FE Grimm KA Kim SA Scanlon KS. Using behavioral risk factor surveillance system data to estimate the percentage of the population meeting us department of agriculture food patterns fruit and vegetable intake recommendations. Am J Epidemiol. (2015) 181:979–88. 10.1093/aje/kwu461 25935424 PMC4465876

[11] Lee SH Moore L V. Park S Harris DM Blanck HM. Adults meeting fruit and vegetable intake recommendations — United States, 2019. Morb Mortal Wkly Rep. (2022) 71:1. 10.15585/mmwr.mm7101a1 34990439 PMC8735562

[12] Vaccaro JA Zarini GG Huffman FG. Cross-sectional analysis of unhealthy foods, race/ ethnicity, sex and cardiometabolic risk factors in U S adults. Nutr Diet. (2018) 75:474–80. 10.1111/1747-0080.12439 29911312

[13] Zeng L Ruan M Liu J Wilde P Naumova EN Mozaffarian D. Trends in processed meat, unprocessed red meat, poultry, and fish consumption in the United States, 1999-2016. J Acad Nutr Diet. (2019) 119:1085–98. 10.1016/j.jand.2019.04.004 31234969 PMC6689198

[14] Daniel CR Cross AJ Koebnick C Sinha R. Trends in meat consumption in the USA. Public Health Nutr. (2011) 14:575–83. 10.1017/S1368980010002077 21070685 PMC3045642

[15] Ajzen I. The theory of planned behavior. Organ Behav Hum Decis Process. (1991) 50:179–211. 10.1016/0749-5978(91)90020-T

[16] Monneuse M Bellisle FGK. Eating habits, food and healthy related attitudes and beliefs reported by French students. Euripean J od Clin Nutr. (1997) 51:46–53. 10.1038/sj.ejcn.1600361 9023467

[17] Nardi VAM Jardim WC Ladeira W Santini F. Predicting food choice: a meta-analysis based on the theory of planned behavior. Br Food J. (2019) 121:2250–64. 10.1108/BFJ-08-2018-050428188865

[18] Wardle J Haase AM Steptoe A Nillapun M Jonwutiwes K Bellisle F. Gender differences in food choice: the contribution of health beliefs and dieting. Ann Behav Med. (2004) 27:107–16. 10.1207/s15324796abm2702_5 15053018

[19] Acheampong I Haldeman L. Are nutrition knowledge, attitudes, and beliefs associated with obesity among low-income hispanic and African American women caretakers? J Obes. (2013). 10.1155/2013/123901 23819044 PMC3681300

[20] Nelson M Ehrens B Bates B Church S Boshier T. Low income diet and nutrition survey. London: TSO, Executive summary. (2007).

[21] Bandura A. Health promotion from the perspective of social cognitive theory. Psychol Heal. (1998) 13:623–49. 10.1080/0887044980840742231295924

[22] McDermott MS Oliver M Svenson A Simnadis T Beck EJ Coltman T. The theory of planned behaviour and discrete food choices: A systematic review and meta-analysis. Int J Behav Nutr Phys Act. (2015) 12. 10.1186/s12966-015-0324-z 26715190 PMC4696173

[23] McEachan R Taylor N Harrison R Lawton R Gardner P Conner M. Meta-Analysis of the Reasoned Action Approach (RAA) to understanding health behaviors. Ann Behav Med. (2016) 50:592–612. 10.1007/s12160-016-9798-4 27169555 PMC4933736

[24] Parkinson J David P Rundle-Thiele S. Self-efficacy or perceived behavioural control: Which influences consumers' physical activity and healthful eating behaviour maintenance? J Consum Behav. (2017) 16:413–23. 10.1002/cb.1641

[25] Guillaumie L Godin G Vezina-Im L-A. Psychosocial determinants of fruit and vegetable intake in adult population: a systematic review. Int J Behav Nutr Phys Act. (2010) 7. 10.1186/1479-5868-7-12 20181070 PMC2831029

[26] Anderson ES Winett RA Wojcik JR. Self-regulation, self-efficacy, outcome expectations, and social support: social cognitive theory and nutrition behavior. Ann Behav Med. (2007) 34:304–12. 10.1007/BF02874555 18020940

[27] Fernández BR Warner LM Knoll N Montenegro EM Schwarzer R. Synergistic effects of social support and self-efficacy on dietary motivation predicting fruit and vegetable intake. Appetite. (2015) 87:330–5. 10.1016/j.appet.2014.12.223 25576023

[28] David P Pennell ML Foraker RE Katz ML Buckworth J Paskett ED. How are previous physical activity and self-efficacy related to future physical activity and self-efficacy? Heal Educ Behav. (2014) 41:573–6. 10.1177/1090198114543004 25156312

[29] McAuley E Szabo A Gothe N Olson EA. Self-efficacy: implications for physical activity, function, and functional limitations in older adults. Am J Lifestyle Med. (2011) 5:361–9. 10.1177/1559827610392704 24353482 PMC3864698

[30] Kendzierski D Costello MC. Healthy eating self-schema and nutrition behavior. J Appl Soc Psychol. (2004) 34:2437–51. 10.1111/j.1559-1816.2004.tb01985.x

[31] Burke PJ. The self: measurement requirements from an interactionist perspective. Soc Psychol Q. (1980) 43:18. 10.2307/3033745

[32] Strachan SM Brawley LR. Healthy-eater identity and self-efficacy predict healthy eating behavior: A prospective view. J Health Psychol. (2009) 14:684–95. 10.1177/1359105309104915 19515684

[33] Dominick JK Cole S. Goals as identities: boosting perceptions of healthy-eater identity for easier goal pursuit. Motiv Emot. (2020) 44:410–26. 10.1007/s11031-020-09824-8

[34] Kendzierski D A. self-schema approach to healthy eating. J Am Psychiatr Nurses Assoc. (2007) 12:350–7. 10.1177/1078390306298983

[35] Bisogni CA Connors M Devine CM Sobal J. Who we are and how we eat: a qualitative study of identities in food choice. J Nutr Educ Behav. (2002) 34:128–39. 10.1016/S1499-4046(06)60082-1 12047837

[36] Dunn KI Mohr P Wilson CJ Wittert GA. Determinants of fast-food consumption. an application of the theory of planned. Behav Appetite. (2011) 57:349–57. 10.1016/j.appet.2011.06.004 21683749

[37] Blake CE Bell BA Freedman DA Colabianchi N Liese AD. The eating identity type Inventory (EITI) development and associations with diet. Appetite. (2013) 69:15–22. 10.1016/j.appet.2013.05.008 23702262 PMC3746737

[38] Jayne JM Frongillo EA Torres-Mcgehee TM Emerson DM Glover SH Blake CE. A healthy eating identity is associated with healthier food choice behaviors among U.S. army soldiers. Mil Med. (2018) 183:e666–70. 10.1093/milmed/usy056 29635635

[39] Gupta NR Freedman DA. Food security moderates relationship between perceived food environment and diet quality among adults in communities with low access to healthy food retail. Public Health Nutr. (2020) (3):1–12. 10.1017/S1368980020001317 32611453 PMC7775890

[40] Carfora V Caso D Conner M. Correlational study and randomised controlled trial for understanding and changing red meat consumption: The role of eating identities. Soc Sci Med. (2017) 175:244–52. 10.1016/j.socscimed.2017.01.005 28129949

[41] Carfora V Caso D Conner M. The role of self-identity in predicting fruit and vegetable intake. Appetite. (2016) 106:23–9. 10.1016/j.appet.2015.12.020 26723237

[42] Lacroix K Gifford R. Reducing meat consumption: Identifying group-specific inhibitors using latent profile analysis. Appetite. (2019) 138:233–41. 10.1016/j.appet.2019.04.002 30965045

[43] Ma X Blake CE Barnes TL Bell BA Liese AD. What does a person's eating identity add to environmental influences on fruit and vegetable intake? Appetite. (2018) 120:130–5. 10.1016/j.appet.2017.08.025 28847565 PMC5680107

[44] Sallis JF Pinski RB Grossman RM Patterson TL Nader PR. The development of self-efficacy scales for healthrelated diet and exercise behaviors. Health Educ Res. (1988) 3:283–92. 10.1093/her/3.3.283

[45] Liu J Kuo T Jiang L Robles B Whaley SE. Food and drink consumption among 1–5-year-old Los Angeles County children from households receiving dual SNAP and WIC v. only WIC benefits. Public Health Nutr. (2017) 20:2478–85. 10.1017/S1368980016002329 27609603 PMC10261461

[46] Moore L V Diez Roux A V Nettleton JA Jacobs DR Franco M. Fast-Food consumption, diet quality, and neighborhood exposure to fast food. Am J Epidemiol. (2009) 170:29–36. 10.1093/aje/kwp090 19429879 PMC2733038

[47] Rosseel Y. lavaan: An R package for structural equation modeling. J Stat Softw. (2012) 48:1–36. 10.18637/jss.v048.i0225601849

[48] Westland JC. Structural equation models: from paths to network. Vol 22. Springer: International Publishing. (2015).

[49] Ferrer-i-Carbonella A Frijtersb P. How important is methodology for the estimates of the determinants of happiness? Econ J. (2004) 114:641–59. 10.1111/j.1468-0297.2004.00235.x

[50] Conner M Kirk SFL Cade JE Barrett JH. Why do women use dietary supplements? The use of the theory of planned behaviour to explore beliefs about their use. Soc Sci Med. (2001) 52:621–33. 10.1016/S0277-9536(00)00165-9 11206658

[51] Brouwer AM Mosack KE. Motivating healthy diet behaviors: the self-as-doer identity. Self Identity. (2015) 14:638–53. 10.1080/15298868.2015.1043335

[52] Gosse MA. How accurate is self-reported BMI? Nutr Bull. (2014) 39:105–14. 10.1111/nbu.12075

[53] McAdams MA Van Dam RM Hu FB. Comparison of self-reported and measured BMI as correlates of disease markers in U. S. Adults Obesity. (2007) 15:188–188. 10.1038/oby.2007.504 17228047

[54] Robinson E Fleming A Higgs S. Prompting healthier eating: testing the use of health and social norm based messages. Heal Psychol. (2014) 33:1057–64. 10.1037/a0034213 24295025

[55] Bleich SN Economos CD Spiker ML Vercammen K VanEpps EM Block JP. A systematic review of calorie labeling and modified calorie labeling interventions: Impact on consumer and restaurant behavior. Obesity. (2017) 25:2018. 10.1002/oby.21940 29045080 PMC5752125

