# Supplementary material for: Associations of Eating Identities With Self-Reported Dietary Behaviors and Body Mass Index
Source: Front Nutr. 2022 Jul 14;9:894557. doi: 10.3389/fnut.2022.894557 (PMC9330157; doi:10.3389/fnut.2022.894557)
Supplement: Supplementary file 1 [file Data_Sheet_1.pdf]

## 1 Supplementary Tables

Table A1. Pearson's r correlations of study's variables

| | 1 | 2 | 3 | 4 | 5 | 6 | 7 | 8 | 9 | 10 | 11 | 12 | 13 | 14 | 15 | 16 | 17 | 18 | 19 | 20 |
|---|---|---|---|---|---|---|---|---|---|---|---|---|---|---|---|---|---|---|---|---|
| 1. Healthy Eater | 1.00 | | | | | | | | | | | | | | | | | | | |
| 2. Meat Eater | -.12* | 1.00 | | | | | | | | | | | | | | | | | | |
| 3. Emotional Eater | -.01 | .20** | 1.00 | | | | | | | | | | | | | | | | | |
| 4. Dietary beliefs | .12* | -.08 | -.05 | 1.00 | | | | | | | | | | | | | | | | |
| 5. Dietary self-efficacy | .18** | -.27** | -.23** | .09 | 1.00 | | | | | | | | | | | | | | | |
| 6. Fruit & veg | .30** | -.09 | .04 | -.02 | .06 | 1.00 | | | | | | | | | | | | | | |
| 7. Water | .16** | .10 | .07 | .09 | -.03 | .20** | 1.00 | | | | | | | | | | | | | |
| 8. Fast food | -.35** | .22** | .08 | -.02 | -.05 | -.11* | -.15** | 1.00 | | | | | | | | | | | | |
| 9. Sugary drinks | -.29** | .19** | .15** | -.01 | -.19** | -.09 | -.11* | .36** | 1.00 | | | | | | | | | | | |
| 10. Sweets | -.24** | .08 | .13* | -.06 | -.12* | .03 | -.06 | .12* | .31** | 1.00 | | | | | | | | | | |
| 11. Fried Foods | -.22** | .16** | .17** | -.03 | -.18** | .04 | .10 | .26** | .38** | .21** | 1.00 | | | | | | | | | |
| 12. BMI | -.34** | .12* | .24** | .12* | -.09 | -.15** | .08 | .20** | .14** | .03 | .12* | 1.00 | | | | | | | | |
| 13. NH White | .11* | -.03 | -.11* | -.08 | .04 | -.04 | -.06 | -.20** | -.31** | .02 | -.18** | -.15** | 1.00 | | | | | | | |
| 14. NH Black | .01 | .13* | .06 | -.05 | .00 | .06 | -.01 | .03 | .03 | -.03 | .05 | .06 | -.24** | 1.00 | | | | | | |
| 15. Hispanic | -.22** | -.09 | -.01 | .10 | -.01 | -.03 | -.03 | .26** | .33** | .07 | .18** | .28** | -.64** | -.23** | 1.00 | | | | | |
| 16. Asian | .08 | .04 | .07 | .04 | .04 | .02 | .06 | -.08 | -.05 | -.04 | -.04 | -.22** | -.25** | -.09 | -.24** | 1.00 | | | | |
| 17. NH Mixed | .12* | .05 | .11* | -.03 | -.11* | .04 | .13* | -.06 | -0.03 | -.11* | -.01 | -.08 | -.19** | -.07 | -.18** | -.07 | 1.00 | | | |
| 18. Age | .18** | -.23** | -.07 | -.03 | .09 | -.05 | -.14** | -.21** | -.39** | -.12* | -.28** | .00 | .46** | -.05 | -.41** | .00 | -.04 | 1.00 | | |
| 19. Male | .10 | .11* | -.05 | -.05 | -.04 | -.15** | -.19** | -.03 | -.15** | -.05 | -.09 | -.01 | .26** | -.10 | -.23** | .04 | -.01 | .25** | 1.00 | |
| 20. College Edu. | .13* | .10 | .00 | -.15** | .03 | .02 | .09 | -.21** | -.29** | -.05 | -.20* | -.26** | .33** | -.10 | -.43** | .16** | .14* | .14** | .24** | 1.00 |
| 21. Birth records | -.08 | .05 | .07 | .10 | -.05 | .10 | .05 | .09 | .31** | .16** | .20** | .09 | -.36** | .04 | .46** | -.13* | -.10 | -.52** | -.34** | -.29** |

*Note.* NH = Non-Hispanic; College Edu. = College Education

*p<0.05; **p<0.01;

[a] *calculated by the average score of the 9 questions evaluated on a scale from 1 to 5;*

[b] *calculated by the average score of the 20 questions evaluated on a scale from 1 to 3;*

[c] *evaluated on a scale from 0 to 3*;

Table A2. Predictors of self-reported sugary drinks consumption; ordinal regression.

| Predictor variables | Dependent variable: self-reported sugary drinks consumption[a] | | | | | |
|---|---|---|---|---|---|---|
| | Model 1<br>Negelkerke $R^2$=0.32<br>ChiSq(11)=120.47*** | | Model 2<br>Negelkerke $R^2$=0.27<br>ChiSq(10)=98.16*** | | Model 3<br>Negelkerke $R^2$=0.32<br>ChiSq(13)=122.23*** | |
| | OR | (95% CI) | OR | (95% CI) | OR | (95% CI) |
| Healthy Eater | **0.61*** ** | **(0.47; 0.77)** | | | **0.63*** ** | (0.49; 0.80) |
| Meat Eater | **1.26*** | **(1.04; 1.51)** | | | **1.23*** | (1.02; 1.48) |
| Emotional Eater | **1.24*** | **(1.04; 1.48)** | | | **1.21*** | (1.01; 1.45) |
| | | | | | | |
| Dietary Beliefs[b] | | | 0.80 | (0.55; 1.18) | 0.91 | (0.62; 1.35) |
| Dietary Self-Efficacy[c] | | | **0.48*** ** | **(0.29; 0.80)** | 0.72 | (0.42; 1.23) |
| | | | | | | |
| <u>Demographics</u> | | | | | | |
| Non-Hispanic White | 0.83 | (0.31; 2.21) | 1.03 | (0.38; 2.74) | 0.90 | (0.34; 2.40) |
| Non-Hispanic Black | 0.87 | (0.27; 2.77) | 1.21 | (0.38; 3.89) | 0.94 | (0.29; 3.02) |
| Hispanic | 1.30 | (0.46; 3.67) | 1.82 | (0.65; 5.11) | 1.46 | (0.51; 4.16) |
| Asian | 0.97 | (0.32; 2.92) | 1.33 | (0.44; 4.05) | 1.09 | (0.36; 3.32) |
| Age | 1.05 | (0.66; 1.67) | 1.05 | (0.67; 1.66) | 1.04 | (0.65; 1.66) |
| Male | **0.96*** ** | **(0.95; 0.98)** | **0.96*** ** | **(0.94; 0.97)** | **0.96*** ** | **(0.95; 0.98)** |
| College Education | **0.41*** ** | **(0.25; 0.68)** | **0.42*** ** | **(0.26; 0.70)** | **0.42*** ** | **(0.25; 0.69)** |
| Recruited through birth records (vs. addresses) | 1.21 | (0.69; 2.14) | 1.06 | (0.61; 1.86) | 1.18 | (0.67; 2.09) |

*Note:* *p<0.05; **p<0.01.*p<0.05; **p<0.01;

[a] *answered on a scale: 0=never, 1=less than once a week, 2=once a week to 3=more than once a week;*

[b] *average response across 9 items on a 1-5 scale;*

[c] *average response across 20 items on a 1-3 scale*

Table A3. Predictors of self-reported sweets consumption; ordinal regression.

| Predictor variables | Dependent variable: self-reported sweets consumption[a] | | | | | |
|---|---|---|---|---|---|---|
| | Model 1<br>Negelkerke $R^2$=0.12<br>ChiSq(11)=40.73*** | | Model 2<br>Negelkerke $R^2$=0.07<br>ChiSq(10)=22.58* | | Model 3<br>Negelkerke $R^2$=0.13<br>ChiSq(13)=41.93*** | |
| | OR | (95% CI) | OR | (95% CI) | OR | (95% CI) |
| Healthy Eater | **0.60*** ** | (0.47; 0.77) | | | **0.62*** ** | (0.48; 0.79) |
| Meat Eater | 1.02 | (0.85; 1.22) | | | 1.00 | (0.83; 1.20) |
| Emotional Eater | **1.27*** | (1.06; 1.52) | | | **1.25*** | (1.04; 1.49) |
| | | | | | | |
| Dietary Beliefs[b] | | | 0.83 | (0.57; 1.21) | 0.91 | (0.62; 1.34) |
| Dietary Self-Efficacy[c] | | | **0.57*** | **(0.35; 0.94)** | 0.77 | (0.46; 1.31) |
| | | | | | | |
| Demographics | | | | | | |
| Non-Hispanic White | **3.11*** | **(1.13; 8.52)** | **3.39*** | **(1.26; 9.13)** | **3.24*** | **(1.18; 8.88)** |
| Non-Hispanic Black | 1.56 | (0.47; 5.11) | 1.84 | (0.57; 6.00) | 1.64 | (0.50; 5.40) |
| Hispanic | 1.86 | (0.64; 5.41 | 2.44 | (0.87; 6.86) | 1.98 | (0.68; 5.78) |
| Asian | 1.95 | (0.61; 6.19 | 2.23 | (0.71; 7.00) | 2.09 | (0.66; 6.66) |
| Age | 1.03 | (0.64; 1.64) | 0.97 | (0.61; 1.52) | 1.02 | (0.64; 1.63) |
| Male | 0.99 | (0.97; 1.01) | 0.99 | (0.97; 1.01) | 0.99 | (0.97; 1.01) |
| College Education | 0.98 | (0.61; 1.58) | 0.92 | (0.58; 1.48) | 0.98 | (0.60; 1.58) |
| Recruited through birth records (vs. addresses) | **1.84*** | **(1.06; 3.20)** | 1.70 | (0.98; 2.93) | **1.83*** | **(1.05; 3.19)** |

*Note:* *p<0.05; **p<0.01.*p<0.05; **p<0.01;

[a] *answered on a scale: 0=never, 1=less than once a week, 2=once a week to 3=more than once a week;*

[b] *average response across 9 items on a 1-5 scale;*

[c] *average response across 20 items on a 1-3 scale*

Table A4. Predictors of self-reported fried-food consumption; ordinal regression.

| Predictor variables | Dependent variable: self-reported fried food consumption[a] | | | | | |
|---|---|---|---|---|---|---|
| | Model 1<br>Negelkerke $R^2$=0.19<br>ChiSq(11)=53.37*** | | Model 2<br>Negelkerke $R^2$=0.15<br>ChiSq(10)=42.79*** | | Model 3<br>Negelkerke $R^2$=0.20<br>ChiSq(13)=57.44*** | |
| | OR | (95% CI) | OR | (95% CI) | OR | (95% CI) |
| Healthy Eater | **0.54*** ** | **(0.38, 0.75)** | | | **0.57*** ** | **(0.41; 0.80)** |
| Meat Eater | 1.17 | (0.91, 1.49) | | | 1.11 | (0.86; 1.43) |
| Emotional Eater | 1.24 | (0.97, 1.58) | | | 1.20 | (0.94; 1.53) |
| Dietary Beliefs[b] | | | 0.71 | (0.42; 1.19) | 0.79 | (0.46; 1.36) |
| Dietary Self-Efficacy[c] | | | **0.37*** ** | **(0.19; 0.71)** | 0.53 | (0.26; 1.09) |
| <u>Demographics</u> | | | | | | |
| Non-Hispanic White | 0.95 | (0.26; 3.48) | 1.33 | (0.35; 5.04) | 1.05 | (0.28; 3.91) |
| Non-Hispanic Black | 1.18 | (0.25; 5.57) | 1.80 | (0.37; 8.84) | 1.34 | (0.28; 6.39) |
| Hispanic | 1.29 | (0.33; 5.10) | 2.11 | (0.52; 8.44) | 1.46 | (0.36; 5.85) |
| Asian | 0.83 | (0.18; 3.74) | 1.19 | (0.26; 5.56) | 0.96 | (0.21; 4.39) |
| Age | 1.07 | (0.58; 2.00) | 1.01 | (0.55; 1.86) | 1.05 | (0.56; 1.95) |
| Male | **0.97*** ** | **(0.94; 0.99)** | **0.96*** ** | **(0.94; 0.98)** | **0.97*** ** | **(0.94; 0.99)** |
| College Education | 0.93 | (0.49; 1.75) | 0.91 | (0.48; 1.72) | 0.92 | (0.48; 1.75) |
| Recruited through birth records (vs. addresses) | 1.28 | (0.60; 2.73) | 1.10 | (0.52; 2.36) | 1.25 | (0.59; 2.68) |

*Note:* *p<0.05; **p<0.01.*p<0.05; **p<0.01;

[a] *answered on a scale: 0=never, 1=less than once a week, 2=once a week to 3=more than once a week;*

[b] *average response across 9 items on a 1-5 scale;*

[c] *average response across 20 items on a 1-3 scale*
